# Variation in use of damage control laparotomy for trauma by trauma centers in the United States, Canada, and Australasia

**DOI:** 10.1186/s13017-021-00396-7

**Published:** 2021-10-14

**Authors:** Derek J. Roberts, Peter D. Faris, Chad G. Ball, Andrew W. Kirkpatrick, Ernest E. Moore, David V. Feliciano, Peter Rhee, Scott D’Amours, Henry T. Stelfox

**Affiliations:** 1 Division of Vascular and Endovascular Surgery, Department of Surgery, University of Ottawa, Room A-280, 1053 Carling Avenue, Ottawa, ON K1Y 4E9 Canada; 2 The Ottawa Hospital Trauma Program, The Ottawa Hospital, Ottawa, ON Canada; 3 School of Epidemiology and Public Health, Faculty of Medicine, University of Ottawa, Ottawa, ON Canada; 4 Clinical Epidemiology Program, The Ottawa Hospital Research Institute, The Ottawa Hospital, Ottawa, ON Canada; 5 The O’Brien Institute for Public Health, University of Calgary, Calgary, AB Canada; 6 Health Services Statistical and Analytic Methods, Data and Analytics (DIMR), Alberta Health Services, Foothills Medical Centre, Calgary, AB Canada; 7 Department of Surgery, University of Calgary and the Foothills Medical Centre, Calgary, AB Canada; 8 Department of Oncology, University of Calgary and the Foothills Medical Centre, Calgary, AB Canada; 9 Regional Trauma Services, Foothills Medical Centre, Calgary, AB Canada; 10 Department of Critical Care Medicine, University of Calgary and Alberta Health Services, Calgary, AB Canada; 11 Department of Surgery, School of Medicine and the Ernest E. Moore Shock Trauma Center at Denver Health, University of Colorado, Denver, CO USA; 12 Department of Surgery and Shock Trauma Center, University of Maryland Medical Center, Baltimore, MD USA; 13 Department of Surgery, Westchester Medical Center, Section of Trauma and Acute Care Surgery, New York Medical College, Valhalla, NY USA; 14 South Western Sydney Clinical School, UNSW, Sydney, NSW Australia; 15 Acute Care Surgery Unit, Liverpool Hospital, Liverpool, NSW Australia; 16 Department of Community Health Sciences, Faculty of Medicine, University of Calgary, Calgary, AB Canada

**Keywords:** Cross-sectional study, Damage control laparotomy, Surgical variation, Wounds and injuries

## Abstract

**Background:**

It is unknown how frequently damage control (DC) laparotomy is used across trauma centers in different countries. We conducted a cross-sectional survey of trauma centers in the United States, Canada, and Australasia to study variations in use of the procedure and predictors of more frequent use of DC laparotomy.

**Methods:**

A self-administered, electronic, cross-sectional survey of trauma centers in the United States, Canada, and Australasia was conducted. The survey collected information about trauma center and program characteristics. It also asked how often the trauma program director estimated DC laparotomy was performed on injured patients at that center on average over the last year. Multivariable logistic regression was used to identify predictors of a higher reported frequency of use of DC laparotomy.

**Results:**

Of the 366 potentially eligible trauma centers sent the survey, 199 (51.8%) trauma program directors or leaders responded [United States = 156 (78.4%), Canada = 26 (13.1%), and Australasia = 17 (8.5%)]. The reported frequency of use of DC laparotomy was highly variable across trauma centers. DC laparotomy was used more frequently in level-1 than level-2 or -3 trauma centers. Further, high-volume level-1 centers used DC laparotomy significantly more often than lower volume level-1 centers (*p* = 0.02). Nearly half (48.4%) of high-volume volume level-1 trauma centers reported using the procedure at least once weekly. Significant adjusted predictors of more frequent use of DC laparotomy included country of origin [odds ratio (OR) for the United States vs. Canada = 7.49; 95% confidence interval (CI) 1.39–40.27], level-1 verification status (OR = 6.02; 95% CI 2.01–18.06), and the assessment of a higher number of severely injured (Injury Severity Scale score > 15) patients (OR per-100 patients = 1.62; 95% CI 1.20–2.18) and patients with penetrating injuries (OR per-5% increase = 1.27; 95% CI 1.01–1.58) in the last year.

**Conclusions:**

The reported frequency of use of DC laparotomy was highly variable across trauma centers. Those centers that most need to evaluate the benefit-to-risk ratio of using DC laparotomy in different scenarios may include high-volume, level-1 trauma centers, particularly those that often manage penetrating injuries.

**Supplementary Information:**

The online version contains supplementary material available at 10.1186/s13017-021-00396-7.

## Background

Damage control (DC) laparotomy was developed to quickly control exsanguinating hemorrhage and gross contamination in injured patients with severe physiologic derangements [1]. It was first adopted by American trauma centers in the 1970s–1990s and then increasingly used worldwide as it was felt to be associated with an increase in unexpected survival among the most critically injured patients [1–4]. However, systematic reviews of randomized and non-randomized studies have found insufficient evidence supporting that use of DC instead of definitive laparotomy improves mortality or other patient-important outcomes [5, 6]. Further, use of the procedure is resource-intensive and associated with increased morbidity when compared to definitive laparotomy [7–13].

The above suggests that there is likely insufficient evidence to support the high DC laparotomy utilization rates reported by some trauma centers [5]. Limited data exist suggests that there is substantial variation in the frequency of use of DC laparotomy across level-1 trauma centers [14–17]. In a post-hoc analysis of the Pragmatic, Randomized Optimal Platelet and Plasma Ratios (PROPPR) trial, DC was used among 33–83% of patients requiring urgent laparotomy across 12 participating American level-1 trauma centers between 2012 and 2013 [17]. While there was no significant mortality difference between the participating trauma centers, the risk of complications was higher among those treated with DC laparotomy [5, 17]. This finding is supported by two other studies which reported that use of DC laparotomy among lower risk cohorts of injured patients is associated with increased risks of complications and longer hospital lengths of stay [5, 16, 18].

Reasons for variation in use of DC laparotomy between level-1 trauma centers in the United States are unknown. It is also unknown whether variation in use of DC laparotomy exists across trauma centers outside of the United States and how often the procedure is used across level-2 and -3 trauma centers (e.g., to stabilize a critically injured patient before transport to a level-1 centers). To address these knowledge gaps, a cross-sectional survey of trauma centers located in the United States, Canada, and Australasia (Australia and New Zealand) was conducted [19]. This survey had two objectives. First, it sought to determine if variation in use of DC laparotomy across trauma centers may be partially driven by surgeon uncertainty as to when the procedure was appropriately indicated. Results of this part of the study have been reported, which suggested that practicing surgeons have relatively consistent impressions of the appropriateness of using DC surgery in certain clinical scenarios [19]. Second, it sought to study variation in the frequency of use of DC laparotomy across level-1, -2, and -3 trauma centers in these regions and predictors of more frequent use of the procedure (the subject of the current study). The study hypothesis was that the reported frequency of use of DC laparotomy would be highly variable across trauma centers, including level-1 trauma centers, and that this variation would be predicted by trauma center and program characteristics, including differences in setting, institutional characteristics, and patient mix.

## Methods

### Design, ethics, and reporting

A self-administered, electronic, cross-sectional survey of trauma program medical directors or leaders located in 4 high-income countries with similar emergency medical services was conducted [19, 20]. Study methods have previously been described in detail [19]. The University of Calgary Conjoint Health Research Ethics Board approved the study. It is reported according to the Strengthening of Observational Studies in Epidemiology (STROBE) statement [21].

### Setting and participants

The population of interest included level-1, -2, and -3 trauma centers that treat adult or adult and pediatric trauma patients in the United States, Canada, and Australasia. The sampling frame of American, Canadian, and Australian trauma centers was identified using lists of those verified by the American College of Surgeons in 2013 [22], that contributed data to the Canadian National Trauma Registry Comprehensive Data Set in 2010 to 2011 (with the exception of Quebec) [23], and that were part of the Australian Trauma Quality Improvement Program as of August 31, 2014 [24], respectively.

### Questionnaire development and testing

The survey questionnaire was developed by modifying a previously developed questionnaire administered to trauma program directors or leaders in the above countries [25]. The modified questionnaire asked for information about respondents’ trauma center, including its geographic location, accreditation/verification, verification level, and academic status. It also asked for information about their trauma program, including the numbers and characteristics of injured patients assessed in the last calendar or fiscal year. Finally, it asked how often the trauma program director estimated DC laparotomy was performed on injured patients at that center on average over the last calendar or fiscal year. This last question had the following ordinal response options: (1) at least once daily, (2) more than once weekly but less than once daily, (3) once weekly, (4) once every 2–3-weeks, (5) once monthly, (6) once every 2–3-months, (7) less than once every 3-months, (8) never, (9) other frequency, or (10) unsure. DC laparotomy was defined in the question stem as “abbreviated laparotomy with planned reoperation (e.g., packing of the liver followed by temporary abdominal closure with plans for reoperation to remove packs at a later time).” The questionnaire’s clarity, length, and completeness were assessed during semi-structured interviews with 5 surgeons or physicians. It was then pilot tested on 5 surgeons or physicians and 2 trauma program directors.

### Questionnaire administration

All trauma centers in the sampling frame were purposively sampled by sending an e-mail to their trauma program director or leader in September, 2014 [26]. E-mails explained the study purpose and invited potential respondents to participate by accessing a link to a Web-based survey. Consent for study participation was inferred with questionnaire completion. To increase response rate, personalized questionnaires were administered that provided assurance of respondent confidentiality [27]. Potential respondents were also sent pre-notification and follow-up e-mails at approximately 1 week, 2–3 weeks, 4–5 weeks, and 5–6 weeks followed by a closing soon e-mail at approximately 7–9 weeks [27].

### Statistical methods

Categorical survey responses were summarized using counts (percentages) and continuous survey responses using medians [with interquartile ranges (IQRs)]. Survey responses were summarized unstratified and stratified by country, reported frequency of use of DC laparotomy, and by volume and level of trauma center care. A high-volume trauma center was defined as per Nathens et al. as one that assessed > 650 major trauma [Injury Severity Scale (ISS) score > 15] patients in the last year [28]. Summary statistics were compared using Fisher’s exact and Kruskal–Wallis tests as appropriate.

Multivariable logistic regression with robust standard errors was used to identify independent predictors of a higher reported frequency of use of DC laparotomy across trauma centers. As there have been no studies to guide selection of evidence-informed predictors for inclusion in the model, all variables felt to be potentially predictive that lacked evidence of multicollinearity were included in the model. We also tested whether the volume of severely injured patients modified the relationship between reported frequency of use of DC laparotomy and level-1 verification status.

The degree of multicollinearity was estimated by calculating associations or correlations between variables and by using the Stata (Stata Corp. College Station, Texas, United States) command package “collin”. Model fit was tested using the Hosmer and Lemeshow’s goodness-of-fit test (non-significant *p* values indicate that the model fits the data). Finally, overall classification performance of the logistic regression model was assessed by generating a receiver operating characteristic (ROC) curve that plotted sensitivity against false-positive rate (1-specificity) across a range of diagnostic thresholds.

Statistical analyses were performed using Stata MP version 13.1.

## Results

### Response rate

Of the 366 potentially eligible trauma program directors or leaders that were sent the survey, 199 (51.8%) responded and provided data on the frequency of use of DC laparotomy in their center.

### Characteristics of participating trauma centers

Of the 199 participating trauma centers, 156 (78.4%) were located in the United States, 26 (13.1%) in Canada, and 17 (8.5%) in Australasia. The 156 participating American trauma centers were located in 37 different states, with most in California (17.3%), Texas (9.6%), and Michigan (8.3%) (see the Figure in Additional File 1). The 26 participating Canadian trauma centers were located in 7 different provinces, with most in Alberta (26.9%), Ontario (26.9%), and British Columbia or Nova Scotia (15.4% each). Finally, of the 17 Australasian trauma centers, 13 (76.5%) were located in Australia and 4 (23.5%) in New Zealand.

Characteristics of the 199 participating trauma centers are outlined in Table 1. Ninety (45.9%) were verified or accredited to provide level-1 trauma care, 72 (36.7%) to provide level-2 care, and 26 (13.3%) to provide level-3 care. Trauma centers in the United States and Australasia assessed more adult (*p* = 0.007) and pediatric (*p* = 0.008) trauma patients than those in Canada. Trauma centers in the United States also assessed a higher percentage of patients with penetrating injuries than those in Canada or Australasia (*p* = 0.008). However, the number of severely injured (defined as an ISS score > 15) patients assessed was similar across trauma centers in the three regions.

**Table 1:** Characteristics of the trauma centers participating in the study

Characteristic (*N* = 199 trauma centers)	No. (%) of trauma centers*			*p* value
	United States (*n* = 156 trauma centers)	Canada (*n* = 26 trauma centers)	Australasia (*n* = 17 trauma centers)	
Accredited/verified for treatment of^a^				< 0.001
Adult patients	119 (76.3)	9 (34.6)	8 (47.1)	
Adult and pediatric patients	34 (21.8)	8 (30.8)	5 (29.4)	
Not accredited/verified—treat adult patients	0 (0)	3 (11.5)	4 (23.5)	
No accredited/verified—treat adult and pediatric patients	3 (1.9)	6 (23.1)	0 (0)	
ACS-designed level of adult care				< 0.001
Level 1	68/154 (44.2)	10/196 (38.5)	12/16 (75.0)	
Level 2	64/154 (41.6)	6 (23.1)	2/16 (12.5)	
Level 3	21/154 (13.6)	4 (15.4)	1/16 (6.3)	
Not accredited/verified	0 (0)	5 (19.2)	1/16 (6.3)	
Other	1/154 (0.7)	1 (3.9)	0 (0)	
Geographic location				0.44
Urban (within a city)	87/150 (58.0)	17 (65.4)	10 (58.8)	
Suburban (residential area on outskirts of a city)	42/150 (28.0)	7 (26.9)	7 (41.2)	
Rural (outside a city)	21/150 (14.0)	2 (7.7)	0 (0)	
Teaching center (regularly has resident physicians on the trauma service)	83/147 (56.5)	17 (70.8)	11 (64.7)	0.39
Participates in research	113/154 (73.4)	22/24 (91.7)	17 (100)	0.006
Local investigator-initiated research	101/154 (65.6)	18/24 (75.0)	13 (76.5)	
Multicenter research	78/154 (50.7)	16/24 (66.7)	11 (64.7)	
Industry-sponsored research	44/154 (28.6)	5/24 (20.8)	5 (29.4)	
Designated trauma team	155/155 (100)	21/25 (84.0)	17 (100)	< 0.001
Designated trauma service	150/154 (97.4)	15/25 (60.0)	14 (82.4)	< 0.001
ICU that admits and cares for injured patients	153/153 (100)	24/24 (100)	17 (100)	NA
No. trauma patients assessed in last year, median (IQR)				
Adult, any ISS score	1500 (953–2524)	836 (650–1349)	1998.5 (1300–3500)	0.007
Adult, ISS score > 15	250 (142–452)	376.5 (129–520)	310 (220–500)	0.67
Pediatric, any ISS score	90 (38–200)	36 (0–100)	68 (20.5–400)	0.008
Pediatric, ISS score > 15	9 (2–27)	6 (0–37)	10 (5–30)	0.84
High volume trauma center^b^	18/135 (13.3)	3/22 (13.6)	1 (6.7)	0.84
Percentage of trauma patients assessed in last year with a penetrating injury, median (IQR)	8 (5–15)	5 (3–9.1)	5 (3–8)	0.008

*ACS* American College of Surgeons, *ICU* intensive care unit, *IQR* interquartile range, *ISS* injury severity scale

^*^Denominator of responses is given if different than stated in the column heading. The number of responses in a category may be greater than the column or category total if responses are not mutually exclusive

^a^Trauma centers in the United States were accredited/verified by the American College of Surgeons; in Canada, the Trauma Association of Canada; and in Australasia, the Royal Australasian College of Surgeons

^b^Defined as a center that assessed > 650 major trauma (ISS > 15) patients in the last year [28]

### Reported frequency of use of DC laparotomy by trauma centers

The reported frequency of use of DC laparotomy for trauma by the 199 participating trauma centers was highly variable (see the Figure in Additional file 2). Forty-four (22.1%) trauma centers reported using DC laparotomy less than once every 3 months. Ten (5.0%) reported never using it. Sixty (30.2%) reported using it once monthly or once every 2–3 months and 73 (36.7%) using it greater than once a month. Twelve (6.0%) trauma centers were unsure how often they used DC laparotomy.

**Fig. 1:**
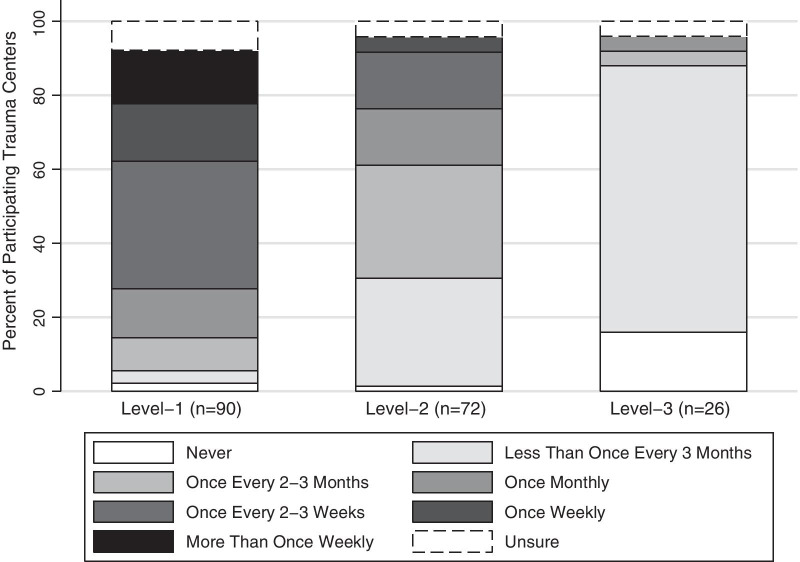
Reported frequency of use of damage control laparotomy for trauma by level-1, -2, and -3 trauma centers in the United States, Canada, and Australasia (Australia and New Zealand)

**Fig. 2:**
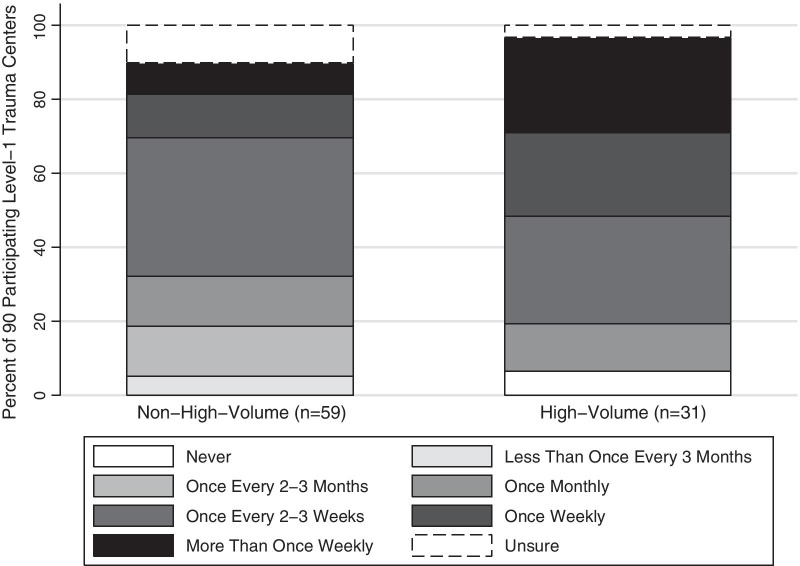
Reported frequency of use of damage control laparotomy for trauma by high- and non-high-volume level-1 trauma centers in the United States, Canada, and Australasia (Australia and New Zealand)

DC laparotomy was reportedly used more frequently in level-1 than level-2 or -3 trauma centers (Fig. 1). High-volume level-1 trauma centers used DC laparotomy significantly more often than lower volume level-1 trauma centers (*p* = 0.02) (Fig. 2). In total, 24 (77.4%) high-volume and 34 (57.7%) lower volume level-1 trauma centers reported using DC laparotomy greater than once monthly. Further, 15 (48.4%) high-volume and 12 (20.3%) lower volume level-1 trauma centers reported using it at least once weekly. Two (6.4%) high-volume level-1 trauma centers reported never using DC laparotomy for trauma.

### Predictors of increased reported frequency of use of DC laparotomy

Characteristics of the trauma centers that reported using DC laparotomy more instead of less frequently are compared in Table 2. Those centers that reported using it more frequently were significantly more likely to be high-volume, level-1, teaching centers located in urban settings. They were also more likely to participate in research and have a designated trauma service. Finally, they were more likely to have assessed a higher number of injured adult and pediatric patients (including those with an ISS > 15) and a higher percentage of patients with penetrating injuries in the last year.

**Table 2:** Characteristics of the participating trauma centers that reported using damage control laparotomy more instead of less frequently

Characteristic (*N* = 199 trauma centers)	No. (%) of trauma centers			*p* value
	Reported using DC laparotomy greater than once monthly (*n* = 73)	Reported using DC laparotomy once monthly or once every 2–3 months (*n* = 60)	Reported using DC laparotomy less than once every 3 months or never using it (*n* = 54)	
Accredited/verified for treatment of^a^				0.17
Adult patients	48 (65.8)	41 (68.3)	39 (72.2)	
Adult and pediatric patients	23 (31.5)	13 (21.7)	8 (14.8)	
Not accredited/verified—treat adult patients	1 (1.4)	3 (5.0)	2 (3.7)	
No accredited/verified—treat adult and pediatric patients	1 (1.4)	3 (5.0)	5 (9.3)	
ACS-designed level of adult care, *n* (%)				< 0.001
Level 1	58 (79.5)	20/59 (33.9)	5/52 (9.6)	
Level 2	14 (19.2)	33/59 (55.9)	22/52 (43.1)	
Level 3	0 (0)	2/59 (3.4)	23/52 (44.2)	
Not accredited/verified or other	1 (1.4)	4/59 (6.8)	2/2 (3.9)	
Geographic location				< 0.001
Urban (within a city)	55/72 (76.4)	28/57 (49.1)	23/52 (44.2)	
Suburban (residential area on outskirts of a city)	13/72 (18.1)	24/57 (42.1)	17/52 (32.7)	
Rural (outside a city)	4/72 (5.6)	5/57 (8.8)	12/52 (23.1)	
Teaching center (regularly has resident physicians on the trauma service)	55 (75.3)	30/55 (54.6)	17/49 (34.7)	< 0.001
Participates in research	64 (87.7)	45/58 (77.6)	33/52 (63.5)	0.006
Local investigator-initiated research	60 (82.2)	39/58 (67.2)	25/51 (49.0)	
Multicenter research	53 (72.6)	24/58 (41.4)	20/51 (39.2)	
Industry-sponsored research	32 (43.8)	17/58 (29.3)	4/51 (7.8)	
Designated trauma team	73 (100)	58 (96.7)	51/52 (98.1)	0.28
Designated trauma service	71 (98.6)	54 (90.0)	46/52 (88.5)	0.04
ICU that admits and cares for injured patients	72/72 (100)	59/59 (100)	51/51 (100)	NA
No. trauma patients assessed in last year, median (IQR)				
Adult, any ISS score	2326 (1552–3034.5)	1300 (953–1897)	733 (480–1081)	< 0.001
Adult, ISS score > 15	449.5 (276–743)	257 (171–400)	97 (50.5–189)	< 0.001
Pediatric, any ISS score	110 (47–360)	86 (39–197)	48 (22–99)	0.009
Pediatric, ISS score > 15	21.5 (4–50)	10 (1–25)	3 (0.5–6)	< 0.001
High volume trauma center^b^	18/66 (27.3)	4/55 (7.3)	0 (0)	< 0.001
Percentage of trauma patients assessed in last year with a penetrating injury, median (IQR)	11.4 (6–17.3)	7.3 (5–10)	4 (2–8)	< 0.001

*ACS* American College of Surgeons, *ICU* intensive care unit, *IQR* interquartile range, *ISS* injury severity scale

^*^Denominator of responses is given if different than stated in the column heading. The number of responses in a category may be greater than the column or category total if responses are not mutually exclusive

^a^Trauma centers in the United States were accredited/verified by the American College of Surgeons; in Canada, the Trauma Association of Canada; and in Australasia, the Royal Australasian College of Surgeons

^b^Defined as a center that assessed > 650 major trauma (ISS > 15) patients in the last year [28]

**Fig. 3:**
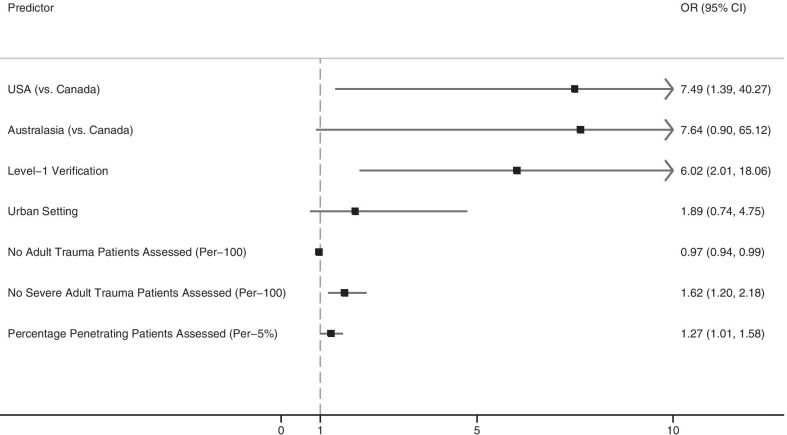
Adjusted predictors of use of damage control laparotomy for trauma more than once a month by trauma centers in the United States, Canada, and Australasia (Australia and New Zealand)

In a multivariable logistic regression model, there was no evidence that the volume of severely injured patients assessed in the last year modified the association between the reported frequency of use of DC laparotomy and level-1 verification status (*p* = 0.35). Variables describing teaching status and trauma center research activities were excluded from logistic regression models because there was evidence of multicollinearity between these variables and level-1 trauma center status. Significant adjusted predictors for more than once monthly reported use of DC laparotomy included country of origin (centers in the United States reported using DC laparotomy significantly more often than those in Canada), level-1 verification status, and the assessment of a higher number of severely injured patients and patients with penetrating injuries in the last year (Fig. 3). The Hosmer and Lemeshow test indicated that the model fit the data well (*p* = 0.62). The area under the ROC curve for the model was 0.88, indicating excellent performance for correctly classifying higher instead of lower reported use of DC laparotomy across trauma centers (see the Figure in Additional file 3).

## Discussion

In this large, international, cross-sectional survey of trauma program directors or leaders, the reported frequency of use of DC laparotomy was highly variable across trauma centers. DC laparotomy was used more often in level-1 than level-2 or -3 trauma centers in the United States, Canada, and Australasia. The frequency of use of DC laparotomy also varied significantly across level-1 trauma centers, especially between those that were high- versus lower volume (based on the number of severely injured patients assessed in the last year). Nearly half of high-volume, level-1 trauma centers reported using DC laparotomy at least once weekly. Trauma center and program characteristics that independently predicted higher reported use of DC laparotomy included country of origin (with centers in the United States reporting using DC laparotomy significantly more often than those in Canada), level-1 trauma verification status, and the assessment of a higher number of severely injured (ISS score > 15) patients and patients with penetrating injuries in the last year.

Studying variation in use of DC laparotomy is important because surgeons are at risk of confirmation bias when only those with whom they work reflect their practice [29]. However, to date, only one other study has examined variation in use of DC surgery between trauma centers [17]. In a post-hoc analysis of the PROPPR trial, DC laparotomy was reportedly used among a highly variable 33–83% of patients requiring urgent laparotomy across 12 American level-1 trauma centers between 2012 and 2013 [17]. In the current study, the trauma program directors who were surveyed also reported that the frequency of use of DC laparotomy varied widely across trauma centers, including level-1 trauma centers. Most level-1 trauma centers reported using DC laparotomy at least once a month, and nearly half of high-volume, level-1 trauma centers reported using it at least once weekly. However, 6% of high-volume, level-1 trauma centers reporting never using DC laparotomy during the past year. Reasons for variation in use of DC laparotomy between level-1 trauma centers may include differences in patient injury mechanisms, injury severity, and/or physiology; trauma center experience; trauma surgeon capabilities; and trauma provider education.

Trauma centers in the United States reported using DC laparotomy for trauma more often than those in Canada despite adjustment for level-1 verification status and the volume of severely injured patients and patients with penetrating injuries. This could be because of differences in institutional cultures regarding use of DC laparotomy between countries. It could also be because of unmeasured differences in patient mix between countries aside from injury mechanism or ISS scores (e.g., a higher percentage of patients with high-risk injury patterns, deranged physiology, or who receive significant volumes of resuscitation fluids) or beliefs regarding appropriate indications for use of the procedure [30]. Of the American, Canadian, and Australasian trauma centers included in this study, a nearly equal percentage reported using the procedure less than once every 3 months or never using it, using it once monthly or once every 2–3 months, or using it more than once monthly. Further, more than one-third of level-2 centers reported using DC laparotomy once monthly or more than once monthly and even some level-3 trauma centers reported using the procedure. We assume that many of these level-3 trauma centers may be using DC laparotomy to stabilize critically injured patients before transport to a higher level of trauma care [7].

In addition to country of origin, other independent predictors of an increased reported use of trauma DC laparotomy included level-1 trauma verification status and the assessment of a higher number of severely injured patients and patients with penetrating injuries in the last year. In the post-hoc analysis of the PROPPR study, the ISS score (OR per-1 point increase = 1.05; 95% CI 1.02–1.07) of the patients assessed at level-1 trauma centers also predicted an increased odds of use of DC laparotomy [17]. This is likely because high-energy blunt torso trauma often results in high ISS scores and also may produce some of the high-risk injury patterns considered by many surgeons to be appropriate indications for DC laparotomy (e.g., massive pelvic fracture-related hemorrhage or multiple injuries spanning across more than one body cavity that each require surgery) [19, 30, 31]. Further, while patients with penetrating injuries may have a lower ISS score, those with gunshot wounds (and especially shotgun wounds) more often present with certain injury patterns that have been suggested to be appropriate indications for DC laparotomy [30]. These may include a major abdominal vascular injury and multiple associated hollow organ injuries or an injured pancreaticoduodenal complex [19, 32].

The study findings should be considered in the context of its strengths and limitations. First, the opinions of trauma program directors could be argued to be only estimates of the frequency of use of DC laparotomy. Surveys are a practical strategy to capture practice variation across hundreds of level-1, -2, and -3 trauma centers in the United States, Canada, and Australasia. However, because of the limitations of using cross-sectional survey data, they should be confirmed by future observational studies. Second, although we used several techniques shown to increase response rates to surveys (and the response rate is above what has been reported by many surveys reported in the trauma or surgery literature), it is possible that respondents’ answers on the reported frequency of use of DC laparotomy differ systematically from those who did not respond to the survey [27]. Third, as the data used in this study are now over 5-years old, it is unclear whether our findings reflect current practice. However, based on evidence that adoption of new evidence-informed practices is slow in medicine (typically quoted to be 18 years), and evolving literature suggesting that deadoption of practices is likely even slower [33, 34], there may have been little change in use of DC laparotomy. This highlights the importance of conducting follow-up studies like ours to track practice patterns and ensure that practice is evidence-informed.

This study has important implications for future research, trauma surgery practice, and quality improvement efforts. First, a systematic review of 36 cohort studies found very little evidence to support that use of DC instead of definitive laparotomy in trauma patients was associated with an improvement in mortality or other patient-important outcomes [5]. However, use of the procedure is associated with an increased risk of morbidity, a longer length of intensive care unit and hospital stay, and possibly a reduced quality of life among survivors and an increase in healthcare utilization [7, 11–13, 35–37]. As equipoise now exists among some surgeons about the effectiveness of DC for improving mortality in many patients undergoing urgent laparotomy, there is a need for rigorously-designed randomized trials comparing it to definitive trauma laparotomy [38]. Second, although DC laparotomy is supported by insufficient evidence, nearly half of high-volume, level-1 trauma centers reported using it at least once weekly. One other study reported utilization rates exceeding 80% in some level-1 trauma centers (with most level-1 centers using it among 30% of those undergoing urgent laparotomy) [17]. Some authors have suggested that more comprehensive indications guiding patient selection for use of DC laparotomy may decrease its associated morbidity and costs [17]. However, our group previously compiled a comprehensive list of indications for the procedure that both experts and practicing trauma surgeons consistently agree appropriately indicate its use [7, 31, 32]. Further, a cohort study suggested that these comprehensive indications that highly predicted use of DC laparotomy in practice had an incidence of 2% or less [30]. Collectively, the above may indicate that DC laparotomy is presently overused in trauma centers. Reasons for this are largely unknown, but may include surgeons’ training, differences in patient mix between institutions, and/or institutional characteristics or culture [19, 29]. However, efforts to decrease use of DC laparotomy across trauma centers may be necessary until further evidence becomes available. The centers that may need to be targeted first include high-volume, level-1 trauma centers, particularly those that often manage penetrating injuries. Indeed, some data suggests that utilization rates of DC laparotomy can be safely reduced through quality improvement efforts such as audit-and-feedback without adversely influencing patient outcomes [14, 39, 40].

## Conclusions

In this large, international, cross-sectional survey of trauma program directors or leaders, the reported frequency of use of DC laparotomy was highly variable across level-1, -2, and -3 trauma centers. The reported frequency of use of DC laparotomy also varied significantly across level-1 trauma centers, especially between those that were high- versus lower volume. The procedure was used most often in level-1 trauma centers in the United States that assessed a large percentage of patients with penetrating injuries. Nearly half of high-volume, level-1 trauma centers reported using DC laparotomy at least once a week. Those trauma centers that most need to evaluate the benefit-to-risk ratio of using DC laparotomy in different scenarios may include high-volume, level-1 trauma centers, particularly those that often manage patients with penetrating injuries.

## Supplementary Information

**Additional file 1.** Location of the 156 participating American trauma centers.

**Additional file 2.** Reported frequency of use of damage control laparotomy for trauma by trauma centers in the United States, Canada, and Australasia (Australia and New Zealand).

**Additional file 3.** Receiver operating characteristic curve for the multivariable logistic regression prediction model.

## Data Availability

Study data are available upon request from the principal author (D.J.R.).

## Abbreviations

CI: Confidence interval
DC: Damage control
IQR: Interquartile range
ISS: Injury severity scale
OR: Odds ratio
PROPPR: Pragmatic, randomized optimal platelet and plasma ratios
ROC: Receiver operating characteristic
STROBE: Strengthening of observational studies in epidemiology
